# Introduction: reporting on updates in the scientific basis for the Lives Saved Tool (*LiST*)

**DOI:** 10.1186/s12889-017-4735-4

**Published:** 2017-11-07

**Authors:** Neff Walker, Ingrid K. Friberg

**Affiliations:** 1 Department of International Health, Johns Hopkins Bloomberg School of Public Health, Baltimore, MD USA; 2 Department of International Public Health, Norwegian Institute of Public Health, Oslo, Norway

The Lives Saved Tool (*LiST*) is designed to support sound public health decisions for women and children. Simply put, it is a computer-based model that draws on best-available evidence to estimate the effect of public health interventions on nutritional status and the causes of mortality among women and children [1]. Model input data include geography-specific demographic information on population, fertility, nutritional status and patterns of mortality by cause and risk factors, the efficacy and – when available – effectiveness of individual interventions, and estimates of the proportion of individuals who need an intervention who actually receive it (coverage). *LiST* has evolved continuously since 2003, when the Bellagio Child Survival Study Group used available coverage data to generate estimates of the child deaths that could be prevented through the achievement of high and equitable coverage with known interventions [2]. This first application led to important changes in priorities for women and children at global and national levels [3]. In the 14 intervening years, *LiST* has changed in important ways, always guided by a commitment to generating and using sound science to improve policies and programs for women and children. The software has been used extensively by national and sub-national programs and by large bilateral and international organizations as part of their planning process for maternal and child health. This supplement – the fourth in an ongoing series [4–6]– makes available the research reports that support these changes and provides the basis for our continuing research and development program in four areas.

## Overview of the supplement

We have divided the 17 papers in the supplement into four broad categories. Each is described below, to provide a rough directory to readers and an overview of the volume.

### LiST assumptions about the efficacy of interventions

*LiST* outputs are only as valid as the input data on which they are based. Estimates of the effect of an intervention – including how an intervention changes the natural history of a specific disease or risk factor and the defined subpopulation affected – are an essential basis for *LiST* calculations. *LiST* collaborators regularly monitor and evaluate new research studies and convene panels of experts to adjust estimates of intervention effects and how they operate in specific disease processes. In principle, *LiST* separates evidence of efficacy (the effect of an intervention under ideal research conditions) from evidence of effectiveness (the effect of an intervention under normal conditions in field settings) [7]. In practice, this is often impossible given that research studies occur in specific temporal, epidemiological and health system contexts that define how an intervention is delivered and received. Guidelines for *LiST* efficacy reviews ask that authors address this tension and report on it transparently [8]. This supplement includes updated efficacy reviews for deworming (Thayer, S13), interventions directed at stillbirths (Blencowe, S8), and the link between diarrhea mortality and interventions for measles vaccination (Jackson, S11) and those intended to improve water, sanitation and handwashing practices (Darvesh, S12).

### Other methodological assumptions

*LiST* outcomes result from hundreds, if not thousands, of subcalculations, using methods and relying on assumptions that reflect a state-of-the-art understanding of health and disease. Promoting peer review of these methods and assumptions is an important mechanism for maintaining scientific integrity, and ensuring that *LiST* continues to reflect best-available science. Previous supplements have explicitly included efforts to assess the internal and external validity of results produced by *LiST*; in this supplement, we broaden this category to include a wider range of methodological investigations. Four reports of recent methodological work are included here. One examines the effects of family planning and other factors on fertility and pregnancy outcomes by comparing *LiST* results to those generated by the broader Spectrum [9] model. (Stover, S6) A second uses ecological methods to predict high-risk births based on contraceptive prevalence and method mix. (Perin, S7) A third explores the potential of using linked household surveys and health facility assessments to develop proxy estimates of intervention coverage. (Kanyagarara, S9) The fourth methods paper in this supplement focuses on how stunting is modeled within *LiST*, and assesses the effect of applying smoothing to linear growth data. (Cousens, S10) Finally, we include a paper that compares and aligns results from *LiST* and the malaria model within Spectrum and offers guidelines to help users reconcile the results generated by the two tools. (Korenromp, S5). Taken together, these five papers provide both important information about the internal and external validity of *LiST* predictions and lay the foundation for further analyses needed to inform public health decisions.

### New and refined tools associated with LiST

The potential of *LiST* to generate useful and often unexpected information has led to the development of new and innovative extensions for specific audiences and applications, often developed in response to repeated requests by users. For example, many users affiliated with intervention- or disease-specific advocacy efforts requested information on estimates of deaths averted through application of just one intervention, or a group of interventions targeting a single disease or risk factor. In response – undertaken in large part to increase the efficiency of responding to these repeated requests – the *LiST* team developed a *LiST*-affiliated tool to produce estimates of the missed opportunities for saving lives attributable to the failure to scale-up one or more individual interventions. (Tam, S2) A second example, illustrated through a multi-country application in this supplement (Clermont, S3), are guidelines for examining the effects of socio-economic inequalities in coverage on maternal and child mortality. Another paper in this supplement responds to the frequent demands by policymakers for estimates of the costs associated with implementing the interventions found by *LiST* to have the greatest potential impact in a particular setting. A new *LiST*-associated module allows users to examine cost estimates and incorporate the results into their efforts to prioritize interventions for implementation. (Bollinger, S4).

### Users and use cases

Much of the information on *LiST* applications is buried in the methods sections of published papers, where authors explain (usually in an annex, if that) how the tool was used to generate their results. Using only published peer-review papers to learn about *LiST* applications introduces a bias, however, toward research applications in preference to those carried out by Ministries of Health and their development partners that rarely appear in print. We try to counter this bias by presenting more descriptions of how *LiST* is being used, including by the UK Government Department for International Development to track the maternal and newborn lives saved from their health-related investments, (Friberg, S14), how the Ministry of Health in Mali used *LiST* to develop their plan for meeting national mortality targets (Keita, S15), and a mixed-method study of *LiST* users carried out by the development team (Stegmuller, S16). The final paper in the supplement builds on these results to examine the challenges of developing a tool that can be used in many different ways, by many different audiences. (Roberton, S17) We encourage all *LiST* users to participate in documenting the experience of using LiST, through participation in our listserv (accessible through the LiST website at http://www.livessavedtool.org).

*LiST* provides an effective mechanism for pushing the science of preventing unnecessary deaths among women and children forward. As reflected in the papers in this supplement, the *LiST* process requires regular reviews of the scientific literature, particularly in areas where there are gaps in our understanding of disease processes and epidemiology, or in methods for the analysis of intervention impact. Because research alone often does not provide a clear or definitive answer, maintaining *LiST* also requires ad hoc consultations with subject matter specialists. The practicalities of modeling require hard thinking and decisions about the best quantitative estimates to include – whether for intervention efficacy, for populations at risk of a disease or likely to benefit from an intervention, or for estimated population coverage. Bringing the best available research reports to the table with leading subject-matter specialists to force a consensus is not easy, and not always successful, but it leads to clarity that we hope leads to better policy decisions and better programs. We thank all the scientists, policy makers and program personnel that contribute to this process.

The need to incorporate new knowledge means that *LiST* is, and must be, in a constant state of development. The moment the tool becomes static – with no new evidence reviews, or no new critical reports of applications – it loses validity and therefore usefulness. We want to thank the Bill & Melinda Gates Foundation for their continued support of the *LiST* Development Team, both financially and technically.

We also thank those who have used *LiST* and participated in the ongoing exchanges of the *LiST* listserv user group. Frank feedback from those who are using the tool provides important guidance for its refinement and improvement.

## Abbreviation

LiST: Lives Saved Tool
